# The role of c-Src in integrin (α6β4) dependent translational control

**DOI:** 10.1186/1471-2121-14-49

**Published:** 2013-11-01

**Authors:** Young Hwa Soung, Nadejda Korneeva, Tae Hyong Kim, Jun Chung

**Affiliations:** 1 Department of Physiology and Stephenson Cancer Center, The University of Oklahoma Health Science Center, Oklahoma City, Oklahoma 73104, USA; 2 Departments of Emergency Medicine and Biochemistry, Louisiana State University Health Science Center, Shreveport, Louisiana 71130, USA; 3 The James Comprehensive Cancer Center, The Ohio State University Wexner Medical Center, Columbus, Ohio 43210, USA

**Keywords:** c-Src, Integrin, Translation, mTOR, VEGF

## Abstract

**Background:**

Integrin α6β4 contributes to cancer progression by stimulating transcription as well as translation of cancer related genes. Our previous study demonstrated that α6β4 stimulates translation initiation of survival factors such as VEGF by activating mTOR pathway. However, the immediate early signaling events that link α6β4 to mTOR activation needs to be defined.

**Results:**

In the current studies, we demonstrated that c-Src is an immediate early signaling molecule that acts upstream of α6β4 dependent mTOR activation and subsequent translation of VEGF in MDA-MB-435/β4 and MDA-MB-231 cancer cells. m^7^GTP-Sepharose–binding assay revealed that Src activity is required to form eIF4F complex which is necessary for Cap-dependent translation in α6β4 expressing human cancer cells.

**Conclusions:**

Overall, our studies suggest that integrin β4 and c-Src activation is important early signaling events to lead mTOR activation and cap-dependent translation of VEGF.

## Background

Cancer cells must acquire survival advantages including growth signaling autonomy, apoptosis resistance, sustaining of angiogenesis under stress conditions such as nutrient and oxygen deprivation to successfully survive in tumor microenvironment [1]. Although these complicated processes involves regulation of survival related gene expression both at the transcription and translational level, recent evidence suggest that translation initiation is a primary check point that regulates cancer related mRNAs [2]. One of the major mechanisms that cancer cells maintain higher efficiency of translation initiation involves stimulation of translation initiation factor, eIF4E [3,4].

eIF4E is the rate limiting factor responsible for delivering cellular mRNAs to eIF4F complex (eIF4E, a scaffold protein eIF4G and a RNA helicase eIF4A) through interaction with the 5’-terminal (m^7^GpppN) Cap structure of mRNAs [5]. Most of the cancer related mRNAs have the highly complex and lengthy 5’ untranslated region, which leads to the low translation initiation efficiency [6]. Therefore, either level or activity of eIF4E needs to be up regulated to maintain active translation of these weak mRNAs. One way to enhance eIF4E activity is through PI3-K/Akt dependent signaling cascade that activates mTOR kinase [7]. Activated mTOR phosphorylates and inactivates eIF4E-binding protein 4E-BP [7]. Upon phosphorylation of 4E-BP, eIF4E is released from 4E-BP and bind to eIF4G to form eIF4F complex which mediates translation initiation [7,8]. Aggressive cancer cells often take advantage of mitogenic signaling pathways to activate mTOR and free up eIF4E to maintain their survival and growth [9-11].

Our previous studies demonstrated that α6β4 integrin stimulates eIF4E activity to promote translation of survival factor, VEGF via Akt/mTOR pathway in breast carcinoma cells under serum deprivation condition [12,13]. While α6β4-dependent translation control via ATK/mTOR pathway has been established, the early signaling event to link between α6β4 and mTOR is not well characterized. One of the prime candidates that mediate α6β4 dependent mTOR activation is Src as it is a key immediate early downstream effector of α6β4 and its activity is required for α6β4 signaling competency [14,15]. Src is an intracellular non-receptor tyrosine kinase which has been implicated in proliferation, metastasis and invasion of various human cancers [16,17]. For example, oestrogen induced c-Src activation leads to 4E-BP phoshorylation through PI3K/mTOR pathway and consequently promotes translation of HIF-1 α in breast cancer cells [18]. Another study showed that active c-Src up-regulates translation of β-catenin by activation of eIF4E via Ras/ERK pathway and the phosphorylation of 4E-BP via the PI3K/mTOR pathways [19] Based on these evidences that c-Src stimulate translational initiation via mTOR signaling, we hypothesized that c-Src mediates α6β4 dependent mTOR activation and subsequent assembly of eIF4E machinery to enhance cap-dependent translation of weak mRNAs.

In this study, we assessed the role of c-Src in α6β4 dependent translational control. Pharmacologic inhibition of c-Src as well as knockdown of its expression by shRNA showed that c-Src plays an essential role in mediating α6β4 dependent mTOR activation in MDA-MB-435/β4 and MDA-MB-231 cancer cells. Src is also required to form eIF4F complex and enhance cap-dependent translation of VEGF mRNA. These results suggest that c-Src is an important immediate early signaling molecule to connect α6β4 signaling to mTOR, which eventually contribute to translation of survival factors such as VEGF.

## Results

### Src activity is required for α6β4 dependent mTOR phosphorylation

**Figure 1:**
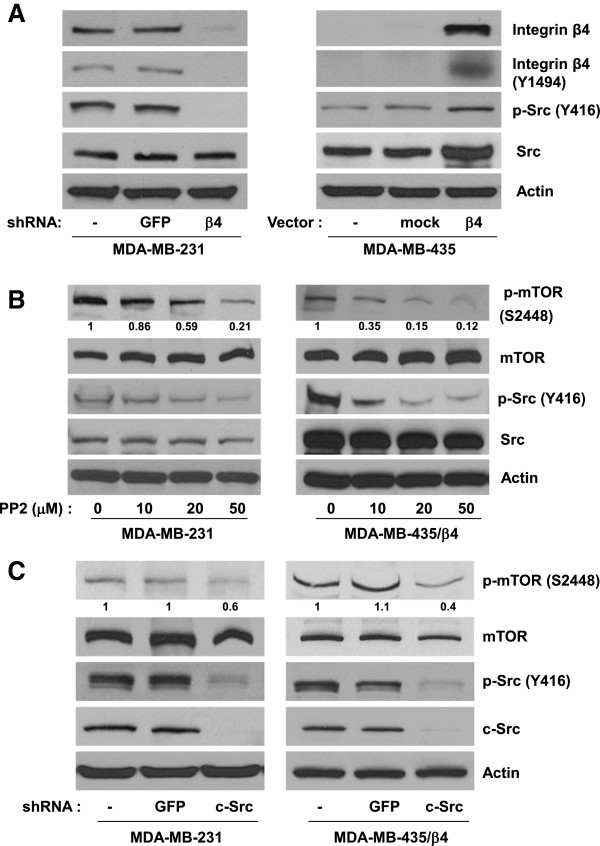
**c-Src mediates α6β4 dependent mTOR activation. (A)** MDA-MB-231breast cancer cells (parental, GFP and β4 shRNA infectants) and MDA-MB-435 human cancer cells (parental, mock and β4 shRNA infectants) were lysed using RIPA buffer. Equal amount of protein were isolated from extracts of these cell lined for Western blot analysis with indicated antibodies. **(B)** MDA-MB-231 cells and MDA-MB-435/ β4 cells were incubated with or without various concentration of PP2 for 24 hr before lysis by RIPA buffer. The laysate were analyzed by Western blotting using antibodies against phospho-mTOR (S2448), mTOR, phospho-Src (Y416), and Src. **(C)**. MDA-MB-231 cells and MDA-MB-435/ β4 cells were infected with lentivirus that stably expresses either GFP or c-Src shRNA. These cells were lysed with RIPA buffer for Western blot analysis with indicated antibodies. The intensity of p-mTOR (S2448) band was quantified by densitometric analysis using ImageJ software. The number given underneath gel image represents fold change compared with control cells. All results are representative of three independent experiments.

α6β4 plays a pivotal role in controlling translation through mTOR signaling [13], but the immediate early signaling events that link α6β4 to mTOR activation remains to be defined. Based on recent reports that c-Src is involved in translation initiation through AKT/mTOR signaling in human cancer cells [18,19], we hypothesized that c-Src is a major mediator for α6β4 dependent mTOR activation. To test this hypothesis, we first assessed the relationship between α6β4 expression and Src activity. We stably knocked down β4 integrin expression in MDA-MB-231 using lentivirus shRNA. MDA-MB-435 cells, which endogenously lack β4 expression, were stably transfected with either β4 integrin or mock vector (Figure 1A). As reported previously by our studies and others [14,20], the reduction of β4 integrin expression by β4 shRNA in MDA-MB-231 cells effectively blocked Src phosphorylation at Y416 (an indicator of Src activation) and β4 phosphorylation at Y1494 (an indicator of β4 integrin activation) (Figure 1A. left panel). The exogenous β4 integrin expression in MDA-MB-435 cells (MDA-MB-435/β4) significantly increased the Src phosphorylation at Y416 (Figure 1A. right panel). We then tested the role of Src in α6β4 dependent mTOR phosphorylation. Pharmacologic inhibition of Src activity by PP2 effectively decreased phosphorylation level of mTOR at Ser2448 (an indicator of mTOR activity) in MDA-MB-231 and MDA-MB-435/β4 cells (Figure 1B). To further confirm the role of Src in α6β4 dependent mTOR phosphorylation, we knocked down expression of c-Src using shRNA in MDA-MB-231 and MDA-MB-435/β4 cells. Knockdown of c-Src expression significantly reduces the level of phosphorylated mTOR at S2448 too (Figure 1C). We were not able to detect a significant change of the total protein level of mTOR by inhibition of Src by PP2 or shRNA. These data suggest that α6β4 dependent c-Src activation leads to the phosphorylation of mTOR.

### c-Src contributes to α6β4 dependent TORC1 and TORC2 activation

**Figure 2:**
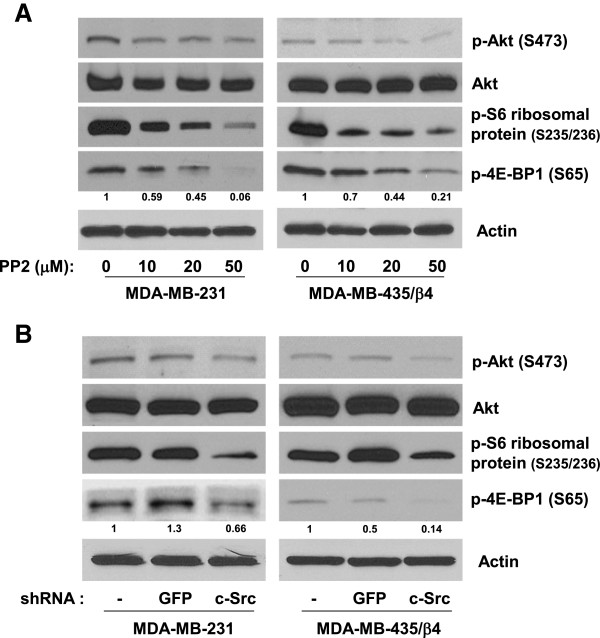
**c-Src is required for α6β4 dependent TORC1 and TORC2 activation. (A)** MDA-MB-231 cells and MDA-MB-435/ β4 cells were incubated with DMSO and indicated concentrations of PP2 for 24 hr before lysis by RIPA buffer. Equal amount of protein were isolated and analyzed by Western blotting using antibodies against phospho-AKT (S473), AKT, phospho-S6 ribosomal protein (S235/236), and phospho-4E-BP1 (S65). **(B)** Equal lysates from MDA-MB-231 cells and MDA-MB-435/ β4 cells expressing shRNA against GFP or c-Src were analyzed by Western blotting with indicated antibodies. The intensity of p-4E-BP1 (S65) band was quantified by densitometric analysis using ImageJ software. The number given underneath gel image represents fold change compared with control cells. All results are representative of three independent experiments.

Mammalian target of rapamycin (mTOR) exists in two functionally and structurally distinct complexes, TORC1 and TORC2 [21]. The primary function of TORC1 is to regulate translation initiation through the phosphorylation of S6K and 4EBP1, whereas the primary function of TORC2 is to regulate survival and proliferation by activation of the kinases such as AKT and SGK [21,22]. To assess relative contribution of c-Src in TORC1 vs. TORC2 activation, we tested the effects of c-Src inhibition on α6β4 dependent Akt phosphorylation at Ser 473 (an indicator of TORC2 activity) and phosphosrylation of S6 ribosomal protein at Ser235/236 and 4E-BP1 at Ser65 (indicators of TORC1 activity) in MDA-MB-231 and MDA-MB-435/β4 cells. Inhibition of c-Src activity by PP2 (Figure 2A) as well as c-Src expression by shRNA (Figure 2B) effectively reduced the level of phosphorylated AKT (on Ser473), S6 ribosomal protein (on Ser235/236) and 4E-BP1 (on Ser65). These results suggest that c-Src mediates α6β4 dependent TORC1 and TORC2 activation.

### Inhibition of c-Src blocks α6β4 dependent translation of VEGF mRNA

**Figure 3:**
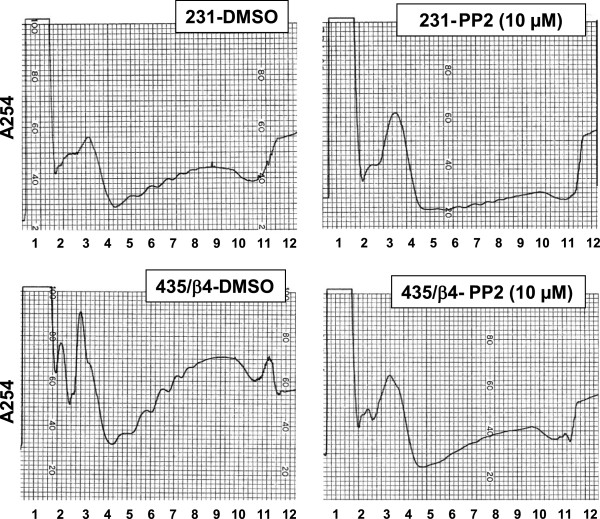
**The effect of Src inhibition on translation initiation.** MDA-MB-231 cells and MDA-MB-435/ β4 cells were treated with DMSO or 10 μM PP2 for 24 hr. Cell lysates were separated into 12 fractions on sucrose gradients as described in the Methods. The distribution was continuously recorded by absorbance at A_254_nm. The polysome profiles were showed as peaks. 1 through 7 represents light polysomes (untranslated mRNA and initiation complex) and 8 through 12 represents heavy polysomes (translated mRNA). Numbers indicates fraction numbers. The results are obtained from three independent experiments.

**Figure 4:**
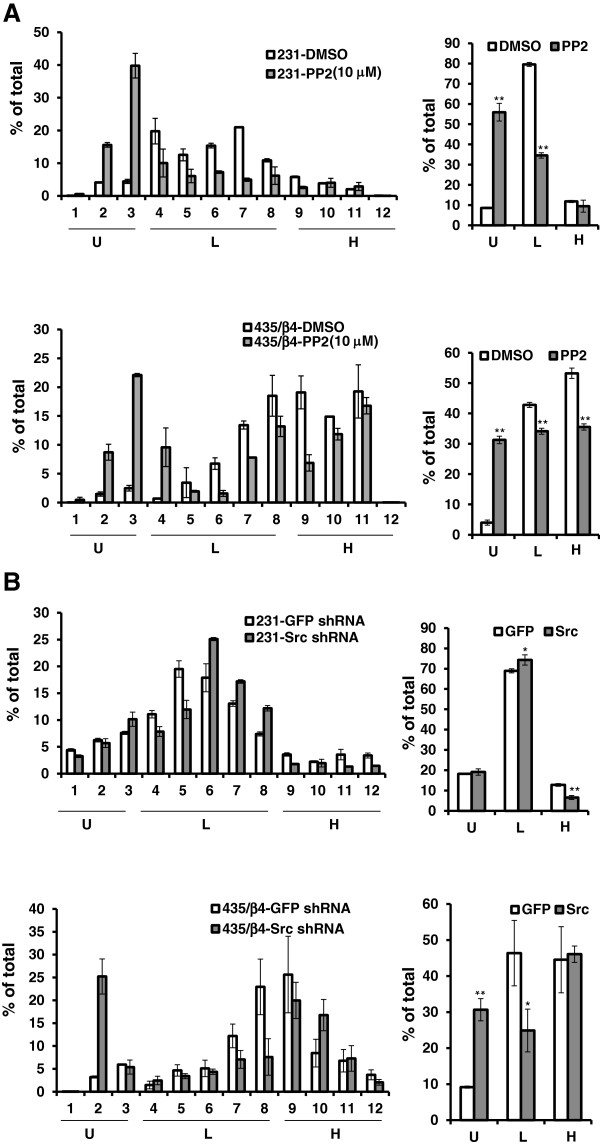
**Src activity is required for α6β4 dependent VEGF translation.** Whole cell lysates were prepared from MDA-MB-231 cells and MDA-MB-435/β4 cells treated with DMSO or 10 μM PP2 overnight **(A)** and infected with GFP or c-Src shRNA **(B)**. The relative VEGF mRNA content of each sucrose gradient fraction was measured by Real time PCR and normalized to GFP mRNA (external control) as described in the Methods. The data are represented as the mean ratio of VEGF to β- actin mRNA (± SD) obtained from triplicate samples. The statistical analysis was done using Student’s *t*-test. * *P* < 0.05, **P < 0.01. U, untranslated complexes (fractions #1-3); L, light polyribosomes (fractions #4-8); H, heavy polyribosomes (fractions #9-12).

We then assessed the effects of c-Src inhibition on the efficiency of overall translation initiation in MDA-MB-231 and MDA-MB-435/β4 cells by performing polysome analysis (Figure 3). The mRNA was isolated from these cells in the presence of either DMSO or PP2, and then fractionated on a sucrose gradient. As shown in Figure 3, the polysome analysis separates untranslated complex (fraction 1 through 3), light polysomes (fraction 4 through 8; poorly translated mRNAs) and heave polysomes (fraction 9 through 12; efficiently well-translated mRNAs). Our previous studies demonstrated that expression of β4 integrin increases the pool of heavy polysomes in these cells [13,21]. The inhibition of Src activity by PP2 dramatically reduced the amount of heavy polysomes (Figure 3), suggesting that Src is required for α6β4 dependent translation initiation. Next, we tested the role of Src in α6β4 dependent VEGF translation. The relative amount of VEGF mRNA in each polysomal fraction was analyzed by qRT-PCR. In the MDA-MB-231 and MDA-MB-435/β4 (Figure 4), VEGF mRNA is distributed mostly in the polysomal region (L and H, fractions #4-12). Both PP2 inhibition of Src activity (Figure 4A) and c-Src knockdown by shRNA (Figure 4B) effectively shifted the distribution of VEGF mRNA to untranslated complexes (U, fractions #1-3). This result indicates that c-Src inhibition affects cap dependent translation initiation of weak mRNAs such as VEGF.

### Inhibition of Src prevents assembly of eIF4F complexes

**Figure 5:**
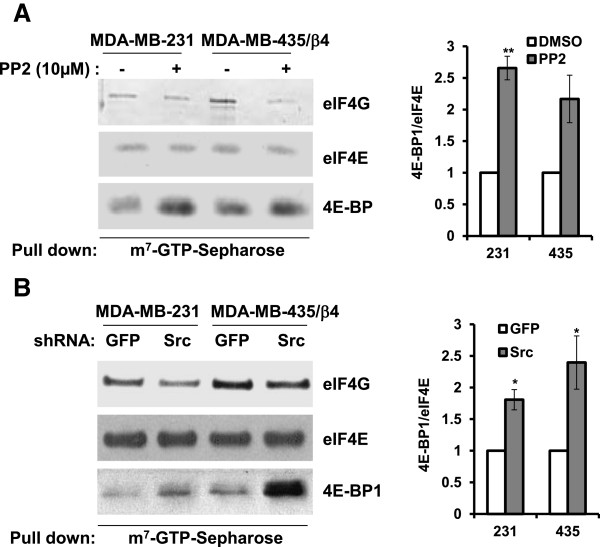
**Src activity is required for eIF-4 F complex.** MDA-MB-231 cells and MDA-MB-435/ β4 cells treated with DMSO or PP2 **(A)** and infected with either GFP- or Src-shRNA **(B)** were lysed and then incubated with m^7^GTP-Sepharose beads. The eluted proteins were analyzed by immunoblotting with antibodies against eIF4G, eIF4E and 4E-BP. The graphs on the right side of each panel represent the mean ratio of 4E-BP1 to eIF4E in triplicate samples (± SD). The statistical analysis was done using Student’s *t*-test. * *P* < 0.05, **P < 0.01.

Since cap-dependent translational efficiency of weak mRNAs such as VEGF is determined by activity of eIF4E and the eIF4F complexes, we examined the role of c-Src in eIF4E-binding to eIF4F components such as eIF4E and eIF4G. We performed m^7^GTP-Sepharose pull down assay in MDA-MB-435/β4 cells to test whether Src inhibition modulates the interaction of eIF4E with eIF4G (an indicator of active translation) or 4E-BP1 (an indicator of inactive translation). The inhibition of Src by PP2 (Figure 5A) and c-Src knockdown by shRNA (Figure 5B) effectively decreased the levels of eIF4G binding to m^7^GTP, whereas the binding level of 4E-BP1 to eIF4E is increased. These data suggests that the inhibition of Src disrupts the assembly of eIF4F complex by inducing the binding of 4E-BP1 to eIF4E, and by disassociating eIF4G from eIF4E.

## Discussion

A number of studies demonstrated the role of integrins in translation of survival and growth factors through enhancing eIF4E function [12], but the exact mechanism by which integrins control translation initiation of cancer related mRNAs remains to be determined. In the previous study, we showed that α6β4 integrin promotes the translation of VEGF mRNA through the AKT/mTOR/eIF4E signaling axis [13]. In the current studies, we investigated the role of c-Src as an immediate early signaling effector that mediates α6β4 dependent mTOR activation. We provided evidence that c-Src inhibition by PP2 or shRNA blocks mTOR pathway and the subsequent assembly of eIF4F complexes. This is first report to define the early signaling event that link between α6β4 and mTOR pathway.

Our studies indicated that c-Src is one of early α6β4 signaling effectors that mediate mTOR activation. As c-Src represents one isoform of Src Family Kinases (SFKs), it is possible that other isoform of SFKs could play a role in α6β4 dependent mTOR activation. This is more likely due to the previous report that Fyn becomes activated to mediate α6β4 dependent pro-invasive migration of breast carcinoma cells [23]. α6β4 dependent Fyn activation requires the recruitment of SHP2 to the phosphorylated cytoplasmic domain of integrin β4 [23]. It remains to be seen whether α6β4 dependent c-Src activation also requires the involvement of SHP2. Another possibility is the involvement of Focal Adhesion Kinase (FAK) in c-Src activation. FAK was shown to be activated by α6β4 [24] and FAK mediates Src activation in integrin signaling such as α5β1 or α4β1 [25]. If we establish the mechanism by which a6b4 activates multiple isoforms of SFKs including Fyn and c-Src, we may need to perform sequential knockdown of each SFK isoform expression by shRNAs to test the role of other SFKs in mTOR activation. The assays will test whether multiple SFK isoform synergistically contribute to α6β4 dependent mTOR activation, or the loss of one SFK isoform could simply be compensated by others.

While our current studies mostly focused on translation initiation aspects of mTOR signaling (mostly through TORC1 pathway), TORC2 pathway is likely activated by α6β4/c-Src signaling axis (Figure 2B). Enhancement of eIF-4E function by α6β4 is known to be mediated by TORC1 pathway as we previously showed that TORC1 specific inhibitor, rapamycin blocked α6β4 dependent eIF-4E activation [13]. It remains to be determined how TORC2 signaling pathway contributes to α6β4 dependent phenotypes of breast carcinoma cells such proliferation, survival, cell motility and invasion. Knockdown of TORC2 specific components such as Rictor or Sin1 [26,27] will address this issue.

It is currently unknown how activated c-Src by α6β4 mediates downstream signaling events leading to mTOR activation. Both Akt and MAPK seem to be prime candidates in mediating c-Src dependent mTOR activation as both involves 4E-BP1 phosphorylation, which is a key event for mTOR activation [19,28]. Activated Src was shown to mediate both Akt [29] and MAPK [30]. Alternatively, c-Src could enhance the functional crosstalk between α6β4 and growth factor receptors such as EGFR and c-Met [31] and this interaction was shown to enhance both Akt [32] and MAPK signaling [33]. All these evidences suggest that c-Src could be an important therapeutic target that could affect growth factor receptor signaling as well as downstream events such as mTOR signaling. Considering that the role of α6β4 in breast carcinoma progression is well established, but no therapeutic agent against α6β4 is available yet, targeting Src activity will merit consideration against tumors that express high levels of α6β4.

## Conclusions

In conclusion, we defined that c-Src is an immediate early signaling molecule that connects α6β4 to mTOR signaling axis. c-Src mediates α6β4 dependent mTOR activation and subsequent enhancement of cap-dependent translation of weak mRNAs such as VEGF. Our finding suggests that c-Src could be an important target of therapy for tumors that express high levels of α6β4.

## Methods

### Cell lines and cultures

The MDA-MB-231 human breast carcinoma cells and MDA-MB-435 human cancer cells were obtained from the Lombardi Breast Cancer Depository at Georgetown University. The generation of MDA-MB-435 subclones (MDA-MB-435/mock (vector only) and MDA-MB-435/β4 (β4 over-expression)) was done as previously described [13]. MDA-MB-231 cells were stably infected with lentivirus that expressed shRNA targeted against β4 integrin or Src and MDA-MB-435/β4 cells were infected against Src as previously described [20]. GFP shRNA was used as control and puromycin (2 μg/ml) was used for the selection of infected cells. Cells were maintained in Dulbecco’s modified Eagle’s medium (DMEM)/low glucose (Hyclone, Logan, UT) supplemented with 10% fetal bovine serum and 1% penicillin-streptomycin (Gibco, Carlsbad, CA).

### Antibodies and reagents

The integrin β4 (clone H-101) and actin (clone C-11) antibodies were purchased from Santa Cruz Biotechnology (Santa Cruz, CA), and the p-mTOR (S2448), p-Src (Y416), p-Akt (S473), p-S6 ribosomal protein (S235/236), p-4E-BP1 (S65), 4E-BP1, mTOR, Src (clone 36D10), and Akt antibodies were obtained from Cell Signaling Technology (Beverly, MA). Also, integrin β4 (Y1494, phospho-specific) antibody was obtained from ECM bioscience (Versailles, KY) and PP2 (Src kinase inhibitor) was purchased from EMD chemicals Inc. (San Diego, CA). The antibodies against eIF4G and eIF4E were kindly provided by Dr. Rhoads (LSUHSC, Shreveport). For the pharmacological inhibition, cells were incubated with or without 10–50 μM PP2 for 24 hours before lysis for Western blot analysis.

### Western blot analysis

Cells were lysed using 50 mM Tris buffer, pH 7.4, containing 150 mM NaCl, 1% NP-40, 0.5% sodium deoxycholate, 0.1% SDS, 1 mM sodium orthovanadate, 5 mM EDTA, 1 mM phenylmethylsulfonyl fluoride, and 1% protease inhibitor (Pierce, Rockford, IL) and scraped, collected, and then protein concentration was determined using BCA protein assay kit (Pierce, Rockford, IL). Total protein was resolved on the 4-20% gradient SDS-PAGE, transferred to polyvinylidene fluoride membranes and incubated with a primary antibody. After three 10 min washes in 50 mM Tris buffer, pH 7.5, containing 0.15 M NaCl and 0.1% Tween-20, protein was detected, in turn, by means of a peroxidase - or alkaline phoaphatase - conjugated secondary antibody and visualized using the Luminol and Oxidizing solutions (Boston Bioproducts, Worcester, MA) or BCIP/NBT Color development substrate (Promega, Madison, WI).

### Ribosome fractionation

The MDA-MB-231 cells and MDA-MB-435/β4 cells were maintained in low serum (0.5% FBS) medium and then pretreated with 0.1% DMSO (as a solvent for PP2) or 10 μM PP2 for 24 h. The MDA-MB-231 cells and MDA-MB-435/β4 cells were infected with lentivirouses expressing GFP- or Src shRNA. Before cell lysis, cells were treated with 50 μg/ml cycloheximide (VWR) and then incubated for 5–10 min at 37°C. After washing with PBS containing 100 μg/ml cycloheximide, cells were lysed in 0.5 ml buffer containing 50 mM Tris–HCl (pH 7.5), 100 mM KCl, 10 mM MgCl_2_, 0.5% NP-40, 2 mM DTT, 100 μg/ml cycloheximide, 50 μg/ml heparin, RNasin 0.5 U/μl (Applied Biosystems), and Complete™ EDTA-free protease inhibitor cocktail (Roche), incubated on ice for 10 min and centrifuged for 5 min at 10,000 × g, 4°C. The supernatants were collected and frozen at -80°C. One hundred μg aliquots of total lysates were used for m^7^GTP-Sepharose binding experiments. An equal volume of lysate was applied to a 15 to 45% (w/v) sucrose gradient containing 100 μg/ml cycloheximide and then centrifuged in a Beckman SW41Ti rotor at 38,000 rpm at 4°C for 3 h. Gradients were fractionated (1 ml) and then monitored for absorbency at 254 nm using an ISCO syringe pump with UV-6 detector.

### RNA preparation and quantitative real time PCR

Before RNA isolation, four hundred aliquots from each fraction after ribosome fractionation were spiked with 100 pg of GFP mRNA (internal control). Then, the RNA was purified from using an E.Z.N.A. Total RNA Kit (Omega bio-tek) according to manufacturer’s instructions. Reverse transcription was performed with random primers and reverse transcriptase from the TaqMan® Reverse Transcription Reagents kit (Applied Biosystems) following the manufacturer’s protocol. Quantitative real time PCR was used to measure the GFP and VEGF mRNAs level in each fraction. Amplification and detection were performed using the iCycler IQ Real-time PCR detection system with IQ™ SYBRgreen Supermix (Bio-Rad). The VEGF mRNA levels were normalized with the GFP internal control. Then, relative amount of VEGF in each fraction was expressed as a percentage of the sum of this mRNA in all fractions. To assist statistical significance of the changes in the VEGF mRNA redistribution along the sucrose density gradients, the percentage of VEGF mRNA co-sedimented with untranslated complexes (U, fractions #1-3), light polyribosomes, containing weakly translated mRNA (fractions #4-8) or heavy polyribosomes, containing efficiently translated mRNAs (H, fractions #9-12), was calculated as a sum of VEGF mRNA in the corresponding fractions from the original data.

### Protein binding assays on m^7^GTP-sepharose

One hundred μg of lysates were prepared as described in the “Ribosome Fractionation” section and then diluted in equal volume of buffer containing 50 mM Tris–HCl (pH 7.5) and 2 mM DTT. The samples were mixed with 50 μl m^7^GTP-Sepharose (GE Healthcare), 50% slurry in buffer containing 20 mM Tris–HCl (pH 7.5), 100 mM KCl, 1 mM DTT, and 10% (v/v) glycerol. After 2 h incubation at 4°C with rotation, the resin was washed three times with 200-μl aliquots of buffer B. Proteins were eluted in 20 μl SDS-electrophoresis buffer and analyzed by Western blotting. To assist statistical significance of the changes in the eIF4E and 4EBP1 binding, the bands of corresponding proteins were scanned and analyzed with ImageQuant TL software.

## Authors’ contribution

YHS prepared for Figures 1, 2 and 3 and wrote introduction, results and methods section. NK prepared for Figures 4 and 5. THK contributed to Figures 1 and 2. JC designed and monitored the whole assays listed in the manuscript, wrote abstract and discussion, and edited the writing in the entire manuscript. All authors reviewed the manuscript. All authors read and approved the final manuscript.
